# Supplementary material for: Are carbon emissions trading and green financial instruments synergistic? -Comprehensive quantitative research based on content analysis
Source: PLoS One. 2024 Mar 7;19(3):e0298601. doi: 10.1371/journal.pone.0298601 (PMC10919601; doi:10.1371/journal.pone.0298601)
Supplement: S1 Data — (ZIP) [file pone.0298601.s001.zip › Data/地级市Z_F工作报告.zip!/║■─╧/╗│╗»╩╨/└┤╫╘╣1⁄2╓┌║┼í╛┬φ┐╦╩2╛▌═°í┐_2014.docx]

各位代表：

现在，我代表市人民政府向大会报告工作，请予审查，并请各位列席人员提出意见。

一、2013年工作回顾

过去的一年，我们在中共怀化市委领导下，坚持以党的十八大精神为指导，认真贯彻落实中央和省委、省政府决策部署，紧紧围绕“构筑商贸物流中心、建设生态文明城市”战略目标，开拓进取，扎实工作，经济社会保持平稳发展。预计全年实现地区生产总值1110亿元，增长10%；完成财政总收入110.47亿元、增长10.12%，公共财政预算收入76.81亿元、增长11.2%；完成固定资产投资800亿元，增长33.3%；完成社会消费品零售总额359亿元，增长13.5%；实现城镇居民人均可支配收入15100元、农民人均纯收入6000元，同口径分别增长9.8%和9.2%。

一年来，我们凝心聚力谋发展，产业结构不断优化。面对经济下行压力加大、要素供应偏紧和特大旱灾等各种挑战，分类施策，主动作为，三次产业结构调整为13.5：44.1：42.4。工业化迈出新步伐。新增规模工业企业12家，规模工业增加值增长10.5%。完成重点工业项目投资75亿元，增长23%。安江水电站并网发电，洪源农林、顺泰科技、金坤碳素、金麦源食品建成投产，金益环保、建南电子、大自然化工、小微企业孵化园等11个亿元以上项目开工建设，本业农机、农网改造等项目进展顺利。怀化工业园、怀化经开区和辰溪、新晃、中方、洪江区工业集中区加快建设，沅陵、会同、靖州、洪江市工业集中区获省规划批复。实施技术创新项目31项，省级企业技术中心增加到6家，开发新产品新技术34项，新增数字企业30家。万元规模工业增加值能耗下降15%，全面完成年度减排目标。现代农业加快推进。全面落实强农惠农政策。完成粮食播面484万亩，总产177.03万吨。杂交水稻制种面积扩大到10.3万亩，成为全国三大杂交水稻制种基地之一。新增油茶基地4万亩、中药材基地8万亩、蔬菜基地3.1万亩，新造工业原料林36.4万亩，新建生猪标准化示范小区13个、肉牛标准化示范小区11个，十大优势农产品基地超额完成年度建设任务。新增农民合作组织447个。建设农业标准化核心园区90个，标准化推广示范面积350万亩。“三品一标”认证产品增加到300个。溆浦、麻阳、洪江市入列农业部柑橘产地初加工补贴范围。市级以上农业产业化龙头企业发展到169家，实现农产品加工销售收入201亿元，增长20.2%。农机总动力达到346.4万千瓦，增长10%。第三产业蓬勃发展。实施重点商贸物流项目32个，完成投资42亿元。佳惠物流配送中心一期、怀化粮食现代物流中心一期和现代农机物流中心、英泰商城、通程百货建成运营。中国丘陵山区首届现代农机·机械展销会、湖南西部首届国际汽博会取得成功。洪江古商城文化旅游区开工建设，黔阳古城获批国家4A景区，靖州飞山和地笋苗寨获批3A景区，通道成功创建全国休闲农业与乡村旅游示范县。举办文化旅游节会活动20余项，实现旅游总收入140亿元，增长26%。竣工商品房475万平方米、销售432.2万平方米，城镇居民人均住房面积达到34.5平方米。金融机构年末各项存款余额达到1120亿元，贷款余额620亿元，存贷比提高到55.4%。

一年来，我们全力以赴抓项目，发展条件持续改善。按照基础先行总要求，着力抓好城乡基础设施建设。提升交通承载能力。实施重点交通项目19个，完成投资143亿元。怀通高速、溆怀高速、G209池回至黔城公路工业园段改线、通道杉木桥至菁芜洲公路建成通车，新增高速公路通车里程284.7公里、干线公路通车里程74.7公里。长昆铁路客运专线怀化段、芷江机场改扩建、新溆高速、怀化绕城高速及在建干线公路如期推进。强力治理车辆违法超限超载，超限运输率由28.5%下降到6%，公路保护得到加强。推进城市提质扩容。启动中心城区提质改造“三年行动计划”，迎丰东路改造二期、怀北路、正清东路、城北入口道路改造、迎丰公园老园区建设顺利完成，市县城市棚户区改造、氵舞水河综合治理、舞阳大道、顺天北路北延、城区供水管网、城镇污水处理配套管网、城镇垃圾处理及中转设施工程完成或超额完成年度投资计划。城市道路交通设施及高清视频监控系统抓紧建设。城区卫生整治、渣土管理、绿化提质、背街小巷改造及县城扩容提质步伐加快。辰溪、新晃成功创建国家卫生县城。全市城镇化率提高到41.2%。加快新农村建设。投入水利建设资金38.5亿元，完成病险水库(水闸)除险加固249座、堤防118公里，治理水土流失面积31.8平方公里，新增和改善灌溉面积26万亩。新建(改造)水源工程562处，解决农村饮水不安全人口35万人。完成农村公路建设956公里、危桥改造52座。建成乡镇客运站44个、招呼站831个。完成国省道南北走廊绿化743公里。新增农村沼气池7200口、卫生厕所2.7万户、太阳能热水器8000户、高效生物质炉灶8000户。麻阳入列国家水土保持生态文明县。

一年来，我们不遗余力破制约，发展活力不断增强。以改革开放、创新创业为动力，着力解决发展中的突出矛盾。积极推进重点领域改革。公路管理体制改革基本完成。交通银行怀化分行开业运营。农村信用社改制稳步推进。组建市林业投资公司，发放林权抵押贷款6亿元。沅陵农村金融服务体系改革试点得到国家部委肯定。麻阳、溆浦农村产权制度改革试点进展顺利。建立农村土地流转服务中心300个，流转土地450万亩。市肉联厂等7家企业改制基本完成。着力推进开放发展。出台加强招商引资工作实施意见、领导干部招商引资绩效考评及奖励办法和委托招商实施办法，启动市投资促进局筹建，举办广州·怀化市情推介会、中国·怀化现代物流产业升级对接会等活动。全年引进产业项目100个，实际到位境外资金9400万美元，增长15%；到位市外境内资金312亿元，增长35.6%。怀化铁路口岸建设获省政府批准。实现进出口总额8800万美元，增长24%。实现境外工程承包1379万元，境外投资354万美元。扎实推动全民创业。制定加快推进全民创业政策，抓好怀化工业园3万平方米创业孵化基地、怀化职业技术学院4000平方米大学生创业基地、市大学生创业园等创业平台建设，市大学生创业园入驻企业达到22家。全市市场主体达到15.1万个。积极推动科技进步。组建特色产业科技创新服务平台，厅市科技工作会商制度完成前期工作。科技项目进入国家计划3个、省级计划97个。授权专利450件。完成高新技术产业增加值60亿元，增长20.9%。

一年来，我们分类指导建小康，县域经济加快发展。认真落实市委四届五次全会精神，建立全面建成小康社会工作机制和考核体系，划分县市区三大类、乡镇街道五大类，召开重点项目现场观摩交流会和“官庄会议”，分类别指导和推进各地工作。突出农村扶贫攻坚，扎实推进100个深度贫困村对口帮扶，引导龙头企业参与产业扶贫，投入项目资金7.76亿元(其中财政专项资金2.49亿元)，实施扶贫项目1513个，覆盖贫困人口73.97万人，直接帮扶贫困农民11.6万人。积极争取上级支持，鹤城和洪江市、洪江区获准“比照执行省级扶贫重点县政策”，沅陵、芷江、新晃、会同、靖州入列国家重点生态功能区补偿范围。10.1万贫困人口稳定脱贫。提升县域经济特色，鼓励和引导各县市区立足实际、突出重点、打造优势，中方县获评“湖南省全面小康经济发展快进县”。县域地区完成生产总值875.4亿元，占全市经济总量的78.9%。

一年来，我们坚持不懈惠民生，各项事业协调发展。

财政民生支出达到175亿元，增长10.8%。20件26项民生实事全面完成。大力发展教育事业。建成义务教育合格学校86所、农村公办幼儿园21所。启动城区教育“第二个五年行动计划”，市二中和顺天路学校改扩建项目交付使用。高中学业水平考试成绩保持在全省第二方阵。怀化职教城建设完成投资1.44亿元。启动怀化医专新校区建设。强化公共卫生服务。市三医院内科大楼、四医院综合医疗大楼及2个县级医院、1个县级急救中心、2个县级卫生监督所、29个乡镇卫生院建设完工。新增县、乡、村卫生项目336个。市中医院和11个县级中医院分别通过三甲、二甲认定。完善新农合运行机制，全市农民参合率提高到97.2%。乡镇卫生院、社区卫生服务中心和95%的村卫生室实现基本药物制度全覆盖。发展文化及其他各项事业。深入开展“欢乐潇湘—幸福怀化”等群众文化系列活动，解决农村电视盲区问题6.2万户，电视综合人口覆盖率达到95%。免费开放市县两级文化馆、图书馆和乡镇综合文化站。文艺创作获得国家级奖项6项。新增全国重点文物保护单位7处。文化经营单位达到1701家。成功举办第四届市运会和首届残运会，群众体育蓬勃发展。加强计生基层基础工作，人口自然增长率控制在7.6‰。抓好耕地保护和用地保障，耕地占补平衡。推进国防教育和国防动员工作，预备役部队和民兵建设水平不断提升。支持工会、共青团、妇女儿童等各项事业发展，统计、人防、档案、供销、水文、气象、地方志、防震减灾、外事侨务、民族宗教、检验检疫、老龄老干、优抚安置、质量技术监督、住房公积金管理等工作取得新成效。不断完善社保体系。新增城镇就业 37094人，农村劳动力转移就业稳定在96万人左右。新增养老、工伤、失业保险参保3.9万人，兑付各项社保待遇37.44亿元。开工建设保障性住房27197套，基本建成21696套。城乡低保覆盖到35万人，月人均补助标准分别提高到264元和120元。新建农村敬老院20所，全市集中供养五保对象7780人。搬迁安置移民1.67万人，实施移民后扶项目647个。下拨救灾资金5140万元，有效应对各类自然灾害。全力维护稳定和谐。深入开展领导干部包案和大接访活动，依法解决群众合理诉求。扎实抓好安全生产“打非治违”专项行动和安全生产大检查，全市生产经营性安全事故下降12.8%、死亡人数下降27.5%。强化食品药品监管，没有发生较大食品药品安全事故。着力净化网络环境，正确引导社会舆论。平安城市和应急指挥体系建设实现年度目标，推动警务工作模式创新，完善“打防控管”一体化社会治安综合治理体系，依法打击违法犯罪活动，社会大局保持和谐稳定。

一年来，我们多管齐下强执行，行政效能得到提升。办复人大代表议案、建议217件、政协委员提案255件，代表、委员满意率99.4%。加强电子政务和电子监察系统建设，网上办结事项90945件，市长信箱办结率99.6%，开通12345市长热线，受理群众信访8120件次，办结率95.6%。推进集中审批服务，市政务中心接待群众69.5万人次，办件受理57.1万件，办结率99.9%，办件上升55%，收费总额下降1倍。清理行政审批项目301项，减少130项，授权怀化工业园市级审批权限77项。开展市直机关“万人评百人”活动和重点项目建设环境“百日清零”整治行动，立案查处影响机关效能和发展环境案件83件。严格执行中央“八项规定”和省委“九项规定”，开展整治“小金库”、治理违规职务消费、清查公务用车、清理办公用房等15项专项治理，查处纠正各类违规违纪问题136起，处理127人，“三公”经费支出零增长。强化政府绩效考核和行政问责，加大审计监察力度，政风行风有新改观。

各位代表，我们能够取得这些成绩，是市委正确领导、科学决策的结果，是市人大、市政协加强监督、支持帮助的结果，是全市人民迎难而上、团结拼搏的结果。在此，我谨代表市人民政府向全市各族人民，驻怀部队、武警官兵、公安政法干警，各民主党派、工商联、人民团体和关心支持怀化发展的各界朋友，表示衷心的感谢!

但是，我们也清醒地认识到，前进道路上还存在不少困难和问题，主要是：我市发展不足、实力较弱、贫困面大的基本市情没有根本改变，全面建成小康社会的任务十分艰巨；经济发展中长期存在的结构性矛盾依然突出，应对宏观环境变化、抵御市场风险能力不强；解放思想、优化环境任重道远，资金、技术等要素制约明显；武陵山片区“同域不同策”问题客观存在，区域竞争压力较大；干部作风不实、行政效能不高、部门执行不力等问题依然存在，等等。对此，我们必须高度重视，切实加以解决。

二、2014年的目标和任务

今年，是全面深化改革的开局之年，是实现“十二五”规划目标的关键之年。政府工作的总体要求是：全面贯彻落实党的十八大及十八届二中、三中全会和中央、省委经济工作会议精神，坚持稳中求进工作总基调，围绕“三量齐升”总要求，按照市委统一部署，把改革创新贯穿于经济社会发展各个领域各个环节，着力激发市场活力，加快调整经济结构，强化基本公共服务，着力保障和改善民生，打好“城镇提质扩容、产业转型发展、扶贫到户到人、机制改革创新”四大攻坚战，促进经济持续健康发展、社会和谐稳定。

主要目标预期是：生产总值增长10%；财政总收入、公共财政预算收入分别增长10%和11%；全社会固定资产投资增长30%；社会消费品零售总额增长13%；规模工业增加值增长12%；居民人均可支配收入增长10%；城镇登记失业率控制在4.5%以内；居民消费价格指数控制在103.5%以下；人口自然增长率控制在8.5‰以内；完成省里下达的节能减排任务。

做好今年工作，必须握紧拳头保重点，认真实施“四个十”工程，即抓好十大产业项目、十大基础设施、十件民生实事、十项重点改革。

十大产业项目：1、托口水电站并网发电；2、华能苏宝顶风电场10台机组装机发电；3、金升阳怀化科技园建成投产；4、怀化商城重建工程主体完工；5、西部汽车产业综合服务中心完成年度投资3.5亿元；6、红星·美凯龙商业广场竣工开业；7、洪江古商城旅游区建设全面实施；8、芷江和平园湖南人民抗战历史陈列馆基本建成；9、奥谱隆怀化种业科技园开工建设；10、完成10万亩杂交水稻制种和120万亩超级稻标准化生产。

十大基础设施：1、长昆客运专线怀化段、怀化南站站前广场竣工；2、新溆高速、绕城高速建成通车；3、芷江机场完成飞行区工程，启动航站楼扩建；4、怀邵衡铁路开工建设；5、G209石羊哨至怀化改造二期竣工；6、中心城区提质改造“三年行动计划”完成年度投资42亿元；7、79个建制镇完成主干道硬化和背街小巷改造。8、114座上型水库水闸除险加固任务全面完成；9、规划建设标准化厂房170万平方米、竣工50万平方米；10、完成高速高铁和国省道绿化提质1323公里。

十件民生实事：1、建立农村扶贫投融资平台，一期授信规模达到20亿元以上；2、完成农民技能培训12万人次，新增农村劳动力转移就业5万人；3、新建农村公路500公里；4、建设农村合格学校101所、教师周转宿舍2636套；5、农村低保扩面提高3个百分点；6、新增创业主体1万个、城镇就业3万人；7、全面完成省政府下达的“两房两棚”、“两供”、“两治”建设任务；8、38个乡镇卫生院建设项目完工；9、解决农村饮水不安全人口29万人；10、推进平安怀化建设，新增社会治安视频监控摄像头1100个。

十项重点改革：1、市直国有商贸企业改革全面完成；2、麻阳农村产权制度改革试点形成经验，全市新增土地流转面积40万亩；3、沅陵农村金融服务体系改革取得新成果，怀化农村商业银行和麻阳农村商业银行挂牌营业；4、深化投融资体制改革，政府性融资平台融资能力和管理水平明显提升；5、推动企业改制上市，本土中小企业进军“新三板”市场融资实现零突破；6、推进城市管理体制改革，完成怀化工业园和经开区管理体制改革；7、深化行政审批制度改革，力争打造全省行政审批事项最少区；8、抓好“营改增”改革试点，推行市直单位财政全口径预算管理；9、加快农村社会化服务体系改革，新增示范性农民合作组织200家以上；10、整合市直部门招商职能，理顺招商体制，创新工作机制。

全年抓好八个方面工作：

(一)以优化结构为主线，加速发展新型工业调整优化产业结构。着眼市场变化和产业升级，大力扶持新能源、新材料、轻工食品、生物医药、节能环保和现代装备制造业。支持用先进装备、技术和工艺改造林纸、矿冶、化工、建材等传统工业，争取开展水电企业直供电试点。积极引进机械、电子等非资源类项目，稀释高能耗企业占比。制订落后产能逐步退出机制，有计划、有步骤地淘汰低端资源加工产品和工艺，全年淘汰水泥立窑生产线6条，关闭小企业10家。

促进产业集群发展。围绕打造百亿园区、千亿产业目标，引导产业向园区集聚。支持骨干企业兼并重组，高水平承接产业转移，推动产业纵向延伸、横向配套，企业专业化分工、集群化发展。按照“工业新城”理念，加大怀化工业园、经开区和各工业集中区水、电、路、气、讯等基础设施建设，把廉租房、公租房等政府资源适当配置到园区，提升园区承载力。继续做好怀化工业园申报国家级园区工作，支持县级工业集中区扩园提质。力争园区工业增加值占全部工业增加值比重提高8个百分点。

培育壮大企业梯队。引导和扶持辰州矿业、大康牧业、骏泰浆纸、大唐华银、金大地、新晃龙脑、汉清生物等骨干企业做大做强，支持有实力的企业上市融资。加强对接央企力度，积极引进大企业大集团。全年新增规模企业23家。落实中小微企业扶持政策，支持有条件的乡镇发展新型工业。健全企业服务体系，改善工业发展条件，促进各类企业茁壮成长。

(二)以转型升级为核心，突出发展现代服务业加速发展商贸物流业。完成现代商贸物流发展规划、流通产业规划编制和城区商业网点规划修编。实施重点商贸物流项目60个、投资50亿元，完成现代粮食物流中心二期、农资物流配送中心、烟草物流中心、佳惠物流配送中心建设，推进博世康医药物流配送中心、隆平国际商业广场、义乌·怀化小商品市场、靖州国际贸易城等重点项目建设,着力打造一批大型卖场、城市综合体、城市商业街区和仓储物流基地。积极引进培育知名商业品牌，促成沃尔玛开业，支持佳惠、怀仁等本土商贸企业扩大市场覆盖，发展总部经济。加快信息平台、结算中心、仓储配送等配套建设，大力发展连锁经营、电子商务等新型业态，促进现代交通体系、物流配送体系、电子商贸体系和现代产业体系对接配套和融合。推进城乡居民商贸服务体系建设，完成农贸市场标准化改造30个，建设“万村千乡市场工程”直营农家店22个。积极发展会展经济，精心筹办汽车展销会、经贸洽谈会等重大节会。

积极发展文化旅游业。迎接高铁时代的到来，加快地方特色文化与旅游业深度融合，务实推动旅游升温。对接周边旅游热点，主动融入张家界-怀化-桂林黄金旅游走廊。抓好黔阳古城、溆浦山背梯田、通道古侗寨、会同粟裕故里、靖州杨梅生态博物馆、沅陵酉水画廊等重点景区景点建设，支持洪江古商城创建4A级景区、通道侗寨申报世界文化遗产和万佛山创建国家地质公园。完善支线机场、高铁高速、干线公路旅游景点标识标牌设置，加快游客集散中心、旅游饭店等配套设施建设，提高旅游接待能力。创新旅游产品宣传促销方式，打造“三古”文化旅游等旅游精品。加快演艺节目、旅游食品、工艺品开发。旅游总收入增长20%以上。

统筹发展其他服务业。加快发展交通运输、教育培训、信息科技等生产性服务业，大力发展宾馆餐饮、家政服务、幼教养老、健康服务、文化娱乐等生活性服务业。支持银行、保险、证券业拓展业务，引导各类金融企业来怀发展，力争长沙银行在怀设立分行，实现农村金融服务中心(站)乡村全覆盖。积极发展各类中介服务机构，规范发展法律、咨询、投资、资产管理等商务服务业。落实国家房地产业政策，加强市场监管和风险防控，支持改善性住房消费。

(三)以全面小康为统领，扎实做好“三农”工作创新农村经营方式。稳定土地承包关系，依法保障农民对承包地占有、使用、收益、流转及承包经营权抵押、担保权利。探索农村土地集体所有制的有效实现形式，落实集体所有权、稳定农户承包权、放活土地经营权，发展家庭农场、专业大户、农民合作社、产业化龙头企业等新型主体，加快构建以农户家庭经营为基础、合作与联合为纽带、社会化服务为支撑的立体式复合型现代农业经营体系。加强土地经营权流转管理和服务，推动土地经营权等农村产权流转交易公开、公正、规范运行。

强力推进精准扶贫。坚持把解决农村贫困人口“两不愁、三保障”、持续推动“六到户”放在突出位置，落实“一乡一策、一村一策、规划到户、扶持到人”工作机制，完成贫困人口精准识别，制订精准扶贫“三年行动计划”。坚持整村推进、对口扶持，抓好100个深度贫困村基础设施建设，用3年时间解决好无房、危房户住房困难。编制完成51个小片区产业扶贫规划，落实国家扶贫资金70%用于产业开发、产业开发资金70%到户到人，鼓励发展“一村一品”经济，力争每个贫困户联结一个农民合作组织或特色产业。抓好金融扶贫，构建金融助推企业、企业带动农户的良性互补扶持模式。引导鼓励生态移民和社会扶贫，大力推进教育扶贫，确保农村贫困孩子不输在起跑线上。全年脱贫人口达到13万人左右。认真落实水库移民后期扶持政策，提高移民生产生活水平。

加快发展现代农业。稳定粮食播面480万亩、总产180万吨左右，加强粮油百亿产业和市县地方粮油储备体系建设，把饭碗牢牢端在自己手上。抓好优质种苗、特色水果基地建设，壮大优势农产品生产规模，抓好“菜篮子”工程，使农民获得更多生产性收入。把农产品质量安全作为转变农业发展方式、加快现代农业建设的关键环节。推进农业标准化提质扩面，实施标准化示范面积350万亩。加强农产品品牌整合创建，新增 “三品一标”农产品认证40个。扶持正清集团、干发茶业、补天药业、惠龙兔业、雪峰米业等龙头企业发展，农产品加工销售收入增长20%。健全农产品质量安全体系，规范农产品检测，加强动植物疫病防控，净化农产品产地环境，确保食品“源头安全”。

提高农业抗灾能力。投资39.8亿元，完成小型农田水利工程建设4万处，新增和改善灌溉面积40万亩，新建和加固堤防112公里，开展107处农村小水电更新改造、10处中小河流治理，推进大中型灌区配套和水源工程建设。加强农业综合开发和耕地质量建设，抓好农村电网改造升级，推进农机应用与服务。做好防汛抗旱、地质灾害防治、气象预警防灾、农业政策性保险、森林防火及病虫害防治等工作。

大力发展县域经济。坚持分类指导，完善促进县域经济发展政策措施，支持县市区依托自身资源，加强项目建设，发展特色优势产业。建立县域经济发展工作推进机制，分期召开旅游、工业、城建现场交流会，加强建制镇、农业乡、相对贫困乡、城关镇、街道办事处工作指导。提升县域经济实力，建设一批经济强镇和产业新镇。

(四)以城镇提质为重点，继续改善基础条件加快城镇基础设施建设。抓好城市总规修编暨鹤中洪芷城镇群规划编制和县城总规、79个建制镇总规修编。推进中心城区提质改造“三年行动计划”，开工建设城区农贸市场改造、断头路拉通工程等37个重点项目，完成城东、城西、杨村高速公路主入口扩改、二环路改造等13大工程建设。抓好舞水河城区段综合治理、太平溪综合治理二三期及清淤补水工程和迎丰、中坡公园建设，推进红星北路、云集路、香洲路、应民北路、四方路、金海路、舞阳大道、平安路二期、正清西路建设。实施城区绿化美化工程，启动城区二套水源、备用水源建设，完善供气供电、停车场、公车、消防、公厕等市政设施，完成215条背街小巷和城中村道路改造。推进人防工程建设，提高城市整体防护能力。加快县城和中心镇建设步伐，拓展新城区，改造旧城区，完善设施配套，优化城市生态，发展城市产业，凝聚人气财气。坚持以人为核心的城镇化，积极推进农业转移人口市民化。加强社会主义新农村建设，完成868个村庄整治规划，引导和支持农民集中建房居住，深入实施农村清洁工程，共同建设幸福家园、美丽乡村。

实施城市精细管理。加快“数字城管·智慧城市”建设步伐，推动城市管理向集约型、精细化、智能化转变。强化城市规划管控，完善城市规划区土地征收和房屋征收补偿安置政策，开展拆违控违、用地清理、打击乱调容积率等专项行动。抓好地下管线规划、建设和管理。规范城区二手车市场，取缔中心集镇马路市场。优先发展城市公交，中心城区新增(更新)公交车50台，建设城区自行车租赁系统。严格渣土运输管理，整治乱搭乱建、乱燃乱放、乱停乱靠等城市乱象。加强城区交通秩序管理，整治车辆非法营运。提高市民素质，提升城市品位，增强城市认同度。

完善城乡交通体系。加快机场、高速高铁、干线公路等在建工程建设，力争武靖高速、怀芷高速、沅辰高速开工建设。抓好S223石门至泸阳段改造工程。推进农村公路通畅工程、连通工程建设。加强运力对接，完善运量、站场、信息、服务配套。抓好湖天北路汽车站、怀化公路客运枢纽站建设。深化治超工作，超限率下降到3%以下。完善公路管理养护机制，确保国省干道、农村公路常年具备良好通畅能力。

(五)以招商引资为抓手，积极发展开放型经济强力推进全民招商。坚持招商引资“第一菜单”理念，强化政绩看发展、发展看项目、项目看招商共识，确保招商引资总额突破420亿元、增长35%以上。突出新型工业、现代服务业、生态农业等产业招商，力争产业投资占投资总额比重达到40%左右。抓实项目前期工作，新增储备项目300个、规模1000亿元以上。创新招商方式，以专业化、产业链、点对点招商为重点，扎实开展亲情招商、以商招商、驻点招商、委托招商和区域市场招商。积极参加国内重大招商节会，充分发挥各类商会、协会、社团及中介组织的作用，多渠道推进招商引资工作。

大力发展外经外贸。加快发展加工贸易，积极开展服务外包业务，优化出口产品结构。巩固传统出口市场，拓展东盟、南美、非洲等新兴市场。加快怀化铁路口岸建设，积极申报海关办事机构，争取保税物流区(仓)落户怀化，对接渝(重庆)新(新疆)欧(欧洲)铁路贸易大通道，主动融入新丝绸之路经济带。加强国际经济技术合作，支持有条件的企业“走出去”，参与国际分工，扩大对外投资、对外工程承包和劳务合作。

加强区域合作和对外交流。着眼湖南“一带一部”区位优势，扩大与长三角、珠三角、长株潭及武陵山片区交流，支持新晃前锋工业园与贵州大龙工业园联手发展，探索区域合作经验。加强对上汇报衔接，拓展厅市共建、银政合作范围和深度，力争全市整体进入国家重点生态功能区补偿范围。做好港澳台侨和外事工作，巩固发展友好城市、友好县区关系，扩大实质性合作。积极为中央和省驻怀单位、高等院校、驻怀部队提供优质服务。加大对外宣传力度，树立对外开放新形象。

(六)以改革创新为动力，不断激发发展活力稳妥推进各项改革。重点抓好国有企业改制、农村改革、财税体制改革、金融体系创新等10项改革，细化“任务书”、“路线图”、“时间表”，务求实效。鼓励发展非公有制经济，积极发展混合型经济，引导国有资本、集体资本、非公有资本等交叉持股、相互融合。注重改革的关联性和耦合性，加强金融生态建设，健全地方政府性债务风险防范机制，规范民间融资，依法打击违规违法金融活动，确保不发生区域性系统性金融风险。

加快推进科技创新。强化企业技术创新主体地位，落实国家支持政策，引导创新要素向企业集中。完善产学研协同创新机制，支持企业与高校、科研院所开展科技合作，推动跨区域跨行业原始创新、集成创新和引进消化吸收再创新。实施一批重点科技专项，开展核心关键技术研究。推进商标兴企兴市战略，培育具有自主知识产权和核心竞争力的名牌产品和企业。加快怀化特色产业科技创新服务平台和武陵山片区生态农业智能控制技术省级重点实验室建设,支持万佛山生态农业科技园申报省级园区。推进专利权质押贷款，探索科技金融结合机制。适应创新需要，建立有利于人才流动的体制机制，大力引进紧缺型、创新型人才，培育创新型领军企业和创新团队。

大力推进全民创业。倡导创业为本、创业为荣、创业为乐的社会风尚，引导支持有创业意愿、创业技能、创业项目的人成功创业。完善创业鼓励政策，放宽经营准入、注册资金、名称登记、经营场所、落户条件限制，推进工商注册制度便利化。整合促进创业就业扶持资金，支持大中专毕业生、转业退伍军人、返乡农民工和失业人员自主创业，鼓励在外怀化籍人士回乡创业。

(七)以美丽怀化为目标，加强生态文明建设加强生态保护和治理。倍加珍惜生态优势。深入实施“碧水青山蓝天”工程，加强林地、湿地、森林公园、风景名胜区、自然保护区保护和建设，巩固发展生态群落。抓好公益林保护、退耕还林、植树造林和采矿沉陷区综合治理，加快沅水、氵舞水等重点流域生态治理，加强饮用水源地保护，构建生态安全圈。抓好疏残林改造和“三边”绿化，推进国省干道、高速高铁沿线通道绿化，建设绿色走廊。新增“三边”人工造林8.5万亩、幼林抚育17万亩、封山育林97万亩。推广农村新能源，新增沼气池5000口、太阳能热水器16500台、省柴灶和高效节能生物质灶7000口。深入推进城乡环境整治，新增农村无害化厕所1万户、卫生厕所3万户。加强重金属和畜禽污染治理，推行医疗废弃物集中处理，开展汽车尾气排放、噪声污染治理和PM2.5监控，加大超标排放处罚力度，提升城乡环境质量。

推进节能减排。加强污水处理厂、污染治理设施运行和重点用能企业监管，推进林纸、化工、建材等传统行业的资源综合利用，强化技术节能和管理节能。抓好城镇污水垃圾处理及供水设施建设专项行动，新建(改造)污水管网111公里、供水管网380公里，新增垃圾处理能力7.5万吨/日。大力倡导绿色消费，引导广大消费者优先选择绿色产品。

集约利用资源。落实最严格的耕地保护和节约用地制度，严守444.3万亩耕地和372.6万亩基本农田红线。加强土地开发整理，有计划地推进低丘缓坡地、未利用地、工矿废弃地综合开发利用，确保耕地占补平衡。推进城区工业“退城入园”，抓好旧城区、城中村、棚户区改造溢余土地收回。严格容积率、投资强度、建筑密度指标控制，推进集约用地。整顿规范土地市场，严格用途管制，严厉打击违法用地。推进石煤、黄金、铅锌、重晶石等矿种资源整合，严格矿权管理，限制和淘汰粗放型资源开采经营项目，加强废弃物综合再利用，提高资源利用效率。

(八)以改善民生为目的，全面推进和谐社会建设协调发展社会事业。推进教育均衡优质发展。实施“安教工程”，用两年时间建好农村教师周转宿舍、消除C、D级危房、完成合格学校建设任务。加强教师队伍和师德师风建设，推进优质教育资源共享。实施城区教育第二个“五年行动计划”，启动宏宇中学、盛世华都小学建设，推进铁一小、铁二中改扩建和顺天路中学、城东幼儿园等项目建设。加快职教基地建设，支持怀化医专升本。抓好市一医院综合楼、妇幼保健院整体搬迁等项目，深化医药卫生体制改革，完善基层医疗卫生服务体系，强化疾病预防控制，提高人民群众健康素质。夯实人口计生工作基层基础，严格整治违法生育，遏制出生人口性别比偏高势头，稳定低生育水平；落实一方是独生子女的夫妇可生育两个孩子政策，促进人口长期均衡发展。积极发展体育事业，广泛开展全民健身运动，做好十二届省运会参赛组织工作。实施文化强市战略，深入开展社会主义核心价值体系宣传教育，抓好重点文化企业、文化项目和基层文化阵地建设，完善公共文化服务。强化文化市场综合执法，促进文化产业健康发展。高度重视民族地区发展，办好通道60周年县庆。加强双拥共建、国防动员、民兵预备役及军事设施保护工作，推动军民融合深度发展。统筹抓好宗教、老龄、台湾事务工作，加大监察、审计工作力度，做好第三次经济普查工作，发展妇女儿童事业，促进地方志、档案、保密、气象、水文、检验检疫等其他各项事业全面协调发展。

创新社会治理机制。强化道德约束、规范社会行为，健全立体化社会治安综合防控体系。推行流动人口居住证“一证通”制度，建立“数字怀化”中心平台，推进治安电子卡口、视频侦查系统等信息化建设，加快公共部位、企事业单位、居民小区等电子监控设备联网。完善基层综合服务管理平台，做好社区矫正工作。依法加大网络管理力度，创新虚拟社会管理。加大基层公共服务投入，保障乡镇(街道)、村组(社区)正常运转，加强基层矛盾纠纷调解中心和社区、农村警务室建设。依法整治突出治安问题，严厉打击各类犯罪。完善矛盾纠纷排查化解、重大事项社会稳定风险评估和群体性事件预防处置机制，加强信访基层基础和网上信访工作，建立涉法涉诉信访依法终结制度。充分发挥工会、共青团、妇联等群团组织的纽带作用，鼓励和支持社会组织参与社会治理。理顺食品药品监管体系，用最严谨的标准、最严格的监管、最严厉的处罚、最严肃的问责，确保食品药品安全。深化安全生产管理体制改革，完善安全生产责任体系，加大安全生产考核权重，切实整改安全生产突出问题，推动农村团寨电改、水改工作，坚决遏制重特大事故发生。

加强民主法制建设。认真落实《法治湖南建设纲要》，积极推进政府自身建设法治化、经济社会管理法治化、利益调整法治化、公共资源配置法治化。自觉执行人大及其常委会的决议决定，接受人大及其常委会的法律监督和工作监督。支持和保障人民政协履行政治协商、民主监督、参政议政职能，认真办理人大代表建议和政协委员提案。推进协商民主广泛多层制度化发展，认真听取民主党派、工商联和无党派人士意见建议，主动接受社会各界、新闻媒体舆论监督，畅通群众监督渠道。大力推进村民自治和城市社区居民自治，保障人民群众依法实行民主选举、民主决策、民主管理和民主监督。深入推进“六五”普法，健全法律援助制度，支持司法机关依法独立行使职权，维护司法公正。

切实保障和改善民生。推动实现更高质量的就业。继续抓好养老、医疗、工伤、生育和失业五大保险，解决好特殊群体参保问题，稳步提高保障水平。完善城乡低保和临时救助制度，确保低收入群体基本生活。关心困难职工生活，维护农民工合法权益。重视空心村问题，建立健全农村留守儿童、妇女、老年人关爱服务体系。推进住房公积金扩面提标，优化公积金提取、使用、监管机制，扩大制度受惠面。发展养老服务产业，健全残疾人、困境儿童社会保障和服务体系，支持发展慈善事业。

三、着力提高行政效能

完成今年工作任务，必须全面加强政府自身建设，扎实开展党的群众路线教育实践活动，增强机遇意识、忧患意识、责任意识，反对形式主义、官僚主义、享乐主义和奢靡之风，规范行政行为，降低行政成本，提高行政效能。

遵循民主依法原则，切实提高行政决策科学性。坚持从群众中来、到群众中去。把民意导向作为工作主调，加强政社政企沟通，推进网络问政、电视问政，办好市长信箱和“12345”市长热线，定期举办网民见面会、新闻发布会和网上互动交流、市民开放日、领导下访坐班接访等活动，做到重大决策顺民而定、工作措施应民而生、发展绩效为民而求。落实政府议事规则，建立健全重大决策公众参与、专家咨询、风险评估、社会听证、法律审查、集体讨论决定工作机制。把组织监督、民主监督、内部监督纳入决策程序，强化决策问责和跟踪纠错。充分尊重经济社会发展规律和地方实际，把年度工作放到怀化发展总体战略中去把握，多做打基础、利长远的实事，多办惠民生、得民心的好事，不搞短期行为，不留发展后患，努力追求没有水分的增长、实实在在的发展、群众满意的政绩。

紧扣规范高效要求，努力打造发展环境优良区。用服务创造有利于各类市场主体积极作为、资源要素竞相集聚的环境。深化行政审批制度改革，推动简政放权，最大限度地减少行政审批事项、减少政府对微观事务的管理。凡是国务院和省政府决定取消或下放的行政审批事项，一律执行到位；凡是有利于增强发展活力、提高运行效率、基层实施更加有效的经济社会管理权限，必须逐级下放。清理压缩行政事业性收费项目，做到公开透明、规范公正。加强诚信政府和诚信怀化建设，弱化部门利益和权力意识，凡是政府承诺了的事项，必须坚决兑现。扩大行政审批和公共服务集中办理范围，规范市政务服务中心运行，推行重点项目全程代办服务，启动网上移动协同办公，完善网上政务服务、电子监察和城乡政务服务体系，深化“万人评百人”活动和依法行政示范单位创建活动，提高行政服务水平和效率。集中整治一批破坏发展环境的突出问题，查处曝光一批典型的人和事。

弘扬少说多干作风，不断增强各级政府执行力。 把重点项目建设作为推动发展的重要抓手，推行项目招商、落地建设、后期服务全程负责制，完善“一个项目、一名领导、一套班子、一个方案、一抓到底”工作机制。全面推行政府工作项目化、项目推进责任化、责任落实具体化，把每一项工作细化为具体项目、量化为具体目标，健全绩效管理、督查督办、定期反馈、办结销号和责任追究制度，确保每项工作落到实处、取得实效。下决心精简会议活动、文件简报，减少公务应酬，下移工作重心，做到在基层一线了解情况、解决问题、推动落实、促进发展。大力弘扬钉钉子精神，重要事项、重大问题、重点工作抓死盯牢，用较真破解发展难题，用实干赢得群众信任，用实绩回报群众支持。

恪守清正廉洁准则，持续提升政府形象公认度。严格执行中央“八项规定”、省委“九项规定”和相关文件精神。全面清理规范公务用车、办公用房，全面规范公务接待，全面停建楼堂馆所，全面推行预算公开和公务卡制度，全面落实行政经费“零增长”、“三公”经费只减不增。逐步推行政府及其工作部门权力清单制度，依法公开权力运行流程，规范基层执法，强化权力制约和监督，促进公平公正。加强对重要领域、重点岗位的行政监察和审计监督，强化招标投标、部门收费、农民减负、质量安全管理、涉农惠农资金等专项治理，严肃查处违法违纪案件。健全改进作风常态化制度，推动为民务实清廉政府建设，以良好的形象取信于民。

各位代表，实干催生巨变，奋斗成就辉煌。让我们在中共怀化市委的坚强领导下，凝聚全市人民的智慧和力量，奋力推进全面建成小康社会进程，共同谱写中国梦的“怀化篇章”!
